# Unique Presentation of Methylenetetrahydrofolate Reductase Mutation With Recurrent Hypercoagulopathy

**DOI:** 10.7759/cureus.85302

**Published:** 2025-06-03

**Authors:** Hiep Nguyen, Siddanth Singh, Shriya Veluri

**Affiliations:** 1 Emergency Medicine, Orthopaedic Surgery, Nova Southeastern University Kiran C. Patel College of Osteopathic Medicine, Fort Lauderdale, USA; 2 Internal Medicine, CHRISTUS Good Shepherd Medical Center, Longview, USA; 3 School of Interdisciplinary Studies, University of Texas at Dallas, Richardson, USA

**Keywords:** anticoagulant therapy, deep vein thrombosis (dvt), homocysteine levels, methylenetetrahydrofolate reductase mutation, submassive pulmonary embolism

## Abstract

Methylenetetrahydrofolate reductase (MTHFR) disease is a disorder in which reduced activity of the MTHFR enzyme disrupts folate and homocysteine metabolism, causing elevated homocysteine levels. Patients with MTHFR mutation suffer many serious lifelong complications. Our case presents a 37-year-old male who was diagnosed with a heterozygous subtype of MTHFR. He suffered multiple life-threatening blood clotting episodes despite being on adequate anticoagulants. His management was challenged by the complexity of his disease progression, as well as limited literature and clinical guidelines on the management of this condition. This unique case report adds to the understanding of clinical manifestations and acute management strategies for patients with heterozygous MTHFR mutations who suffer excessive clotting as a complication.

## Introduction

Methylenetetrahydrofolate reductase (MTHFR) disease is a disorder that causes impaired folate and homocysteine metabolism. This is most commonly due to improper transcription of a gene, resulting in reduced enzymatic activity and lower levels of homocysteine. MTHFR is an enzyme responsible for many bodily functions involving vitamin B9, also known as folate. This enzyme converts the inactive form of B9 (folic acid) into the active form of folate and finally 5-methylenetetrahydrofolate (CH3-THF), which is the major form of folate in the human body. CH3-THF, in turn, converts homocysteine to methionine via methionine synthase using vitamin B12 as a cofactor. Improper transcription of the MTHFR gene can lead to various pathologies, including anencephaly, spina bifida, homocystinuria, and other genetic disorders [1]. It has also been shown to increase the risk of specific malignancies, cardiovascular disease, blood clots, and pulmonary embolisms. Here, we present a rare case report of heterozygous MTHFR mutation complicated by recurrent hypercoagulopathy episodes despite appropriate anticoagulation, resulting in a complex hospital course and clinical management. 

## Case presentation

A 37-year-old Caucasian male with a past medical history of superior mesenteric vein thrombosis currently on apixaban (Eliquis®), deep vein thrombosis, acute lower gastrointestinal (GI) bleeding, anemia, and depression presented to the emergency room with a chief complaint of persistent shortness of breath and right leg pain. The patient stated that walking even a short distance resulted in shortness of breath. Other associated symptoms include dizziness, presyncope episodes, chest tightness, and generalized weakness. The patient denied loss of consciousness, fever, or focal neurological deficits.

In the emergency room, the patient was noted to be afebrile, tachycardic, tachypnic, and saturating 100% on room air, with a body mass index (BMI) of 33. Home medications include apixaban, fluoxetine, hydroxyzine, methocarbamol, pantoprazole, sucralfate, and trazodone. Physical examination showed bilateral leg swelling; the remainder of the exam was unremarkable. Labs were concerning for elevated prothrombin time of 18 seconds (normal: 9.9s-14.9s), high partial thromboplastin time of 42.4 seconds (normal: 25.1s-36.5s), and internationalized normal ratio of 1.5. platelets were also elevated at 510 x 103/µl (normal: 150 x 103/µl - 450 x 103/µl).

**Figure 1:**
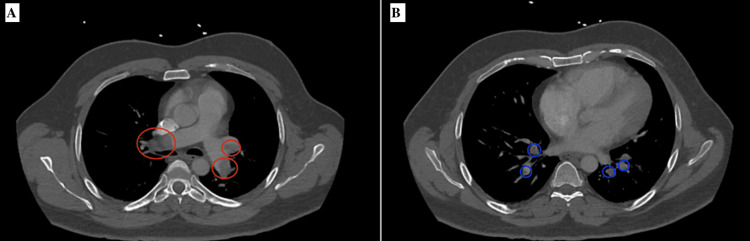
CT chest showing massive emboli in the right and left pulmonary arteries (Figure 1A, red circles) and scattering emboli in multiple pulmonary arteries (Figure 1B, blue circles).

Computed tomography angiography (CTA) of the chest during this admission showed repeated massive emboli in the right and left pulmonary arteries, along with scattered thromboembolic burden bilaterally, more pronounced on the right lower lobe pulmonary artery and extending into several segments (Figure 1). Vascular Doppler ultrasound of bilateral lower extremity veins demonstrated thrombosis of the right posterior vein and left popliteal and femoral veins. Further review of the patient's medical record revealed that he was diagnosed with heterozygous MTHFR. Genetic testing was performed at that time and showed a heterozygous mutation of the C677T variant. At the time of his diagnosis, his homocysteine level was in the high normal range at 14 umol/L (normal 0-14) and elevated factor VIII at 315 (50-200). 

**Figure 2:**
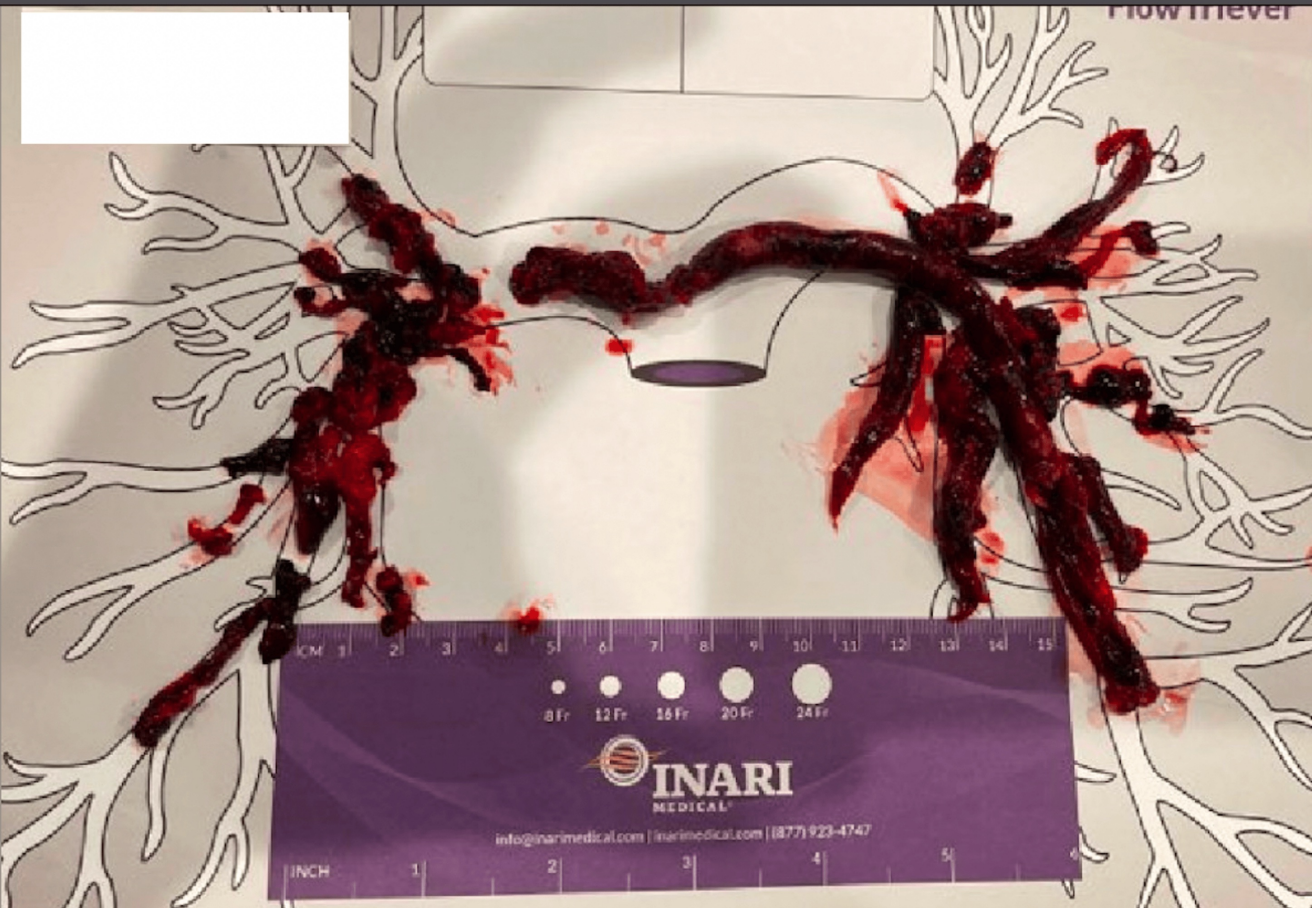
Extensive emboli clotting the pulmonary arteries removed with thrombectomy.

Prior medical records showed that he was hospitalized 10 days prior for bilateral deep vein thrombosis (DVT) and submassive pulmonary embolism (PE) throughout the entire pulmonary vascular system with right heart strain (Figure 2). He was treated with a thrombectomy and discharged on Eliquis 5 mg twice daily, bridging from heparin. A few days later, the patient was hospitalized again for DVT despite being compliant with anticoagulation and had an inferior vena cava filter placed for pulmonary embolism (PE) prophylaxis.

Cardiology and hematology were consulted for recurrent PE and deep vein thrombosis (DVT). Based on prior outpatient records from his hematologist-oncologist, the patient was negative for protein C or S deficiency, factor V Leiden mutations, the factor II G20210A mutation, antithrombin III levels, antiphospholipid syndrome, malignancy, and disseminated intravascular coagulation. A decision was made to treat the patient with the EkoSonic Endovascular System (EKOS) (Bothell, Washington, United States) catheter infusion of tissue plasminogen activator. The patient reported improving symptoms after the procedure. Repeat CTA chest showed improved PE. He was then placed on a heparin drip as the bridge to warfarin for his long-term coagulation therapy upon discharge once the international normalized ratio (INR) became therapeutic. 

## Discussion

MTHFR is an uncommon hematologic disease with a vast diversity of complications. There is significant existing literature on the clinical manifestations of homozygous MTHFR mutations, which include hydrocephalus, motor abnormalities, brain atrophy and demyelination, seizures, and motor and other psychiatric conditions [2]. However, very little information has surfaced on the complications and disease course of the heterozygous subtype, leading to difficulty in treating patients like the individual presented in this case. Specifically, his presentation of excessive clotting despite adequate direct oral coagulant highlights the unique manifestation. This case report adds to existing literature by describing a possible clinical presentation and acute clinical management of patients with heterozygous MTHFR mutations, although further research is required to fully understand additional clinical manifestations and long-term disease progression.

**Figure 3:**
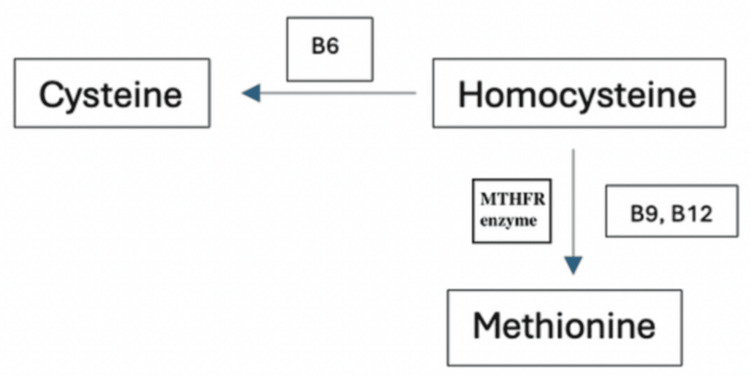
Homocysteine metabolism pathway. The image is created by the author.

MTHFR mutation interrupts the conversion of homocysteine to methionine. The MTHFR enzyme plays an important role in the metabolism of folate, which is an important cofactor for the synthesis of methionine from homocysteine (Figure 3). Elevated homocysteine levels due to an unfunctional MTHFR enzyme result in endothelial dysfunction, which can be linked to cardiovascular disease [3]. The inheritance pattern of this mutation is autosomal recessive, with the most common variants associated with this condition being C677T and A1298C mutations [4]. Studies suggest that 30-40% of the population carries one copy of the mutated gene, given the nature of the autosomal recessive inheritance pattern. This population does not exhibit the phenotypic manifestation, making this disease more difficult to diagnose. The prevalence is not significantly associated with any particular racial or ethnic group.

Patients with MTHFR mutation have a significantly increased risk for hypercoagulopathy conditions, such as DVT and PE. It is estimated that homozygous MTHFR variants have a moderate risk for venous thromboembolism (VTE); this risk increases to 50% of cases with compound mutation [5]. Our patient’s factor VIII level was elevated, which could be an effect modifier for his recurrent thrombi formation. Regardless, standard anticoagulation therapy remains the cornerstone of treatment for thromboembolism. Heparin and warfarin, as well as direct oral anticoagulants (DOACs) like rivaroxaban and apixaban, are commonly used. In emergency cases, such as hemodynamic instability, thrombolytic therapy can be used to treat PE [6,7]. Vena cava filters may be inserted in specific situations where anticoagulation is contraindicated or ineffective [8].

As for treating MTHFR, there is very limited data on effective treatment options. One can theorize that there may have been an error for the patient to metabolize his anticoagulation. Treatment failure with apixaban can result from various factors that compromise its efficacy. One significant cause is drug-drug interactions, particularly with medications that affect cytochrome P450 enzymes or P-glycoprotein transporters, which can alter apixaban's plasma concentration. Additionally, patient-specific factors such as renal or hepatic impairment can influence drug metabolism and clearance, leading to subtherapeutic levels. Non-adherence to the prescribed dosing regimen is another critical factor, as missed doses can reduce anticoagulant effects and increase the risk of thromboembolic events. Furthermore, genetic polymorphisms affecting drug-metabolizing enzymes may also contribute to variability in patient response to apixaban [9].

Overall, based on the clinical symptoms and workup as well as the patient’s medical history, a heterozygous MTHFR mutation was determined to be the culprit for increasing this patient's risk of hypercoagulability. He was initially on Eliquis after being treated for his previous DVT and PE; however, this did not prevent additional hypercoagulopathy episodes. Common differential diagnoses to consider alongside MTHFR mutation include factor V Leiden mutation, protein C or S deficiency, antiphospholipid syndrome, or prothrombin deficiency, and other conditions include malignancy, disseminated intravascular coagulation (DIC), obesity, or use of estrogen therapy [10]. Because this patient’s workup was negative for all the above-mentioned conditions, the MTHFR mutation was ultimately established as the underlying cause for the increasing risk of hypercoagulability in this patient.

## Conclusions

To the best of our knowledge, this is a unique case presentation that demonstrates a correlation between MTHFR gene mutation and the risk of developing a hyperthrombolic state. Although common risk factors for VTE and PE should be addressed, one should not dismiss the examination for genetic causes, particularly when there is a recurrence. After the likely cause of clotting is identified, further treatment options can be pursued to determine the greatest efficacy. Finally, patient satisfaction and adherence to treatment should improve given an explanation for their signs and symptoms.
